# Synergistic antibacterial activity of surfactant free Ag–GO nanocomposites

**DOI:** 10.1038/s41598-020-80013-w

**Published:** 2021-01-08

**Authors:** Muhammad Ashfaq Ahmad, Samia Aslam, Faiza Mustafa, Usman Arshad

**Affiliations:** Department of Physics, COMSATS University Islamabad, Lahore Campus, Lahore, 54000 Pakistan

**Keywords:** Materials science, Nanoscience and technology, Physics

## Abstract

Graphene oxide–silver (Ag–GO) nanocomposite has emerged as a vital antibacterial agent very recently. In this work, we report a facile one step route of Ag–GO nanocomposite formation excluding the aid of surfactants and reductants and was successfully applied to negative *Escherichia Coli* (*E coli*) to investigate antibacterial activity by varying doze concentration. The successful formation of Ag–GO nanocomposite via facile one step route was confirmed using Fourier transform infrared spectroscopy (FTIR) and Raman Spectroscopy. The absorption spectra (peak ~ 300 nm) for GO and the (peak ~ 420 nm) for silver nanoparticles were observed. XRD study confirmed the formation of Ag–GO nanocomposite while atomic force microscopy (AFM) showed crumbled GO sheets decorated with Ag nanoparticles. It was observed that the functional groups of GO facilitated the binding of Ag nanoparticles to GO network and enhanced the antibacterial activity of the nanocomposite.

## Introduction

Graphene and its derivatives have attracted enormous interest because of their promising electrical, electronic, optical, thermal, mechanical properties and versatile bio-medical applications. Graphene Oxide (GO) is a 2-dimensional nanomaterial packed into dense honey-comb lattice structure having reactive functional groups of oxygen that allow nanoparticles to interact with GO without the need of functionalization^1,2^. Recent reports suggest that GO and related materials are biologically compatible holding negligible or no cytotoxicity whereas its hydrophilic nature dictates its role as an ideal carrier and transporter of nanoparticles as antimicrobial agents^3^.

Silver (Ag) nanoparticles have been the primary nanoparticles to be used for antimicrobial activities. Their medicinal applications involve reduction in inflammation infections, bandages formulations, filtration systems and in textiles^4^. The Ag nanoparticles interact with bacterial cells thus inhibiting the cellular respiration followed by division of the cell causing its death^5^. However, it is reported by researchers^6^ that bare Ag nanoparticles are prone to their agglomeration in solutions which affects their stability and thus antibacterial activity. Recently, it has been demonstrated by various researchers that GO sheets can be used to functionalize Ag nanostructures and effectively prevent their agglomeration in dispersions. Ag–GO nanocomposite formation which has proved a synergistic antibacterial activity with excellent biocompatibility^7,10^.

Many previous and recent researchers have reported the formation of Ag–GO nanocomposite using additional surfactants including chitosan^11^ polyethylene analyine (PEI)^12^, green tea leaves^13^, Cetyl trimethylammonium bromide (CTAB)^14^, Silane ligand^15^ and reductants^11–14^ to improve stability and aggregate prevention. However, these materials may introduce further limitations including interference factors and may also introduce potential environmental and toxicity factors^12^. Moreover, they also increase the experimental steps and may make the synthesis time intensive. In scenario of the ongoing risen interest of researchers in Ag–GO nanocomposite formation and studying its antibacterial applications, there is a room to investigate surfactant and reductant free facile methods of its formation and studying whether its antibacterial activity is altered by this approach, which to our knowledge have not been so far studied for antibacterial activities.

Instigated by this approach, we therefore report in this work, a facile, cost effective surfactant and reductant free method for successful Ag–GO formation and investigated its anti-bacterial activity on gram-negative bacteria *Escherichia coli* (*E. coli*) as a model. Whereas we observed synergistic inhibiting growth rate of the microbes showing parity with surfactant functionalized Ag–GO nanocomposites reported in literature mentioned above.

## Experimental section

### Synthesis of materials

Graphene oxide was prepared by modified Hummer’s method. Briefly, 1 g of graphite powder was mixed with 27 ml of sulfuric acid (H_s_SO_4_) (Sigma Aldrich) and 0.5 g of silver nitrate (AgNO_3_) (Alfa Aeser) and stirred, for 30 min in an ice bath. After that, 3 g of potassium permanganate (KMNO_4_) (Sigma Aldrich) was added slowly to the mixture under stirring. The reaction was mediated by adding 1000 ml of distilled water followed by slowly adding 30 ml of 30% H_2_O_2_ (Pancreas). The sample was later washed several times with distilled water and diluted Hydrochloric acid (HCl) (Sigma Aldrich) until the pH was neutralized to 7.

### Antibacterial study

The antibacterial activity of Ag–GO was evaluated by using gram-negative bacteria *Escherichia coli* (*E. coli*) cultured in LB-broth as the test strains. The incubation was performed at 37 °C under shaking at 150 rpm.

### Characterization

The absorption spectrum of Ag–GO was examined over the range of 200–700 nm using UV/Vis/NIR Spectrophotometer (PerkinElmer LAMBDA 750). Fourier transform infrared (FTIR) spectra were obtained on a Nicolet 6700 spectrometer (Thermoscientific, USA) in the range of 4000 to 500 cm^−1^. X-ray diffraction (XRD) was carried out using Philips PANalytical—X-ray diffractometer using $Cu-k\alpha (\lambda =1.54\, \AA )$. The Raman spectra was obtained on In Via RAMAN Microscope, Renishaw, UK (Raman & PL set up), with a 514 nm green (Argon) laser light by applying $1\%$ intensity of the full laser power (20 mW) to avoid the sample damage.

## Results and discussion

### UV–Vis spectroscopy

**Figure 1:**
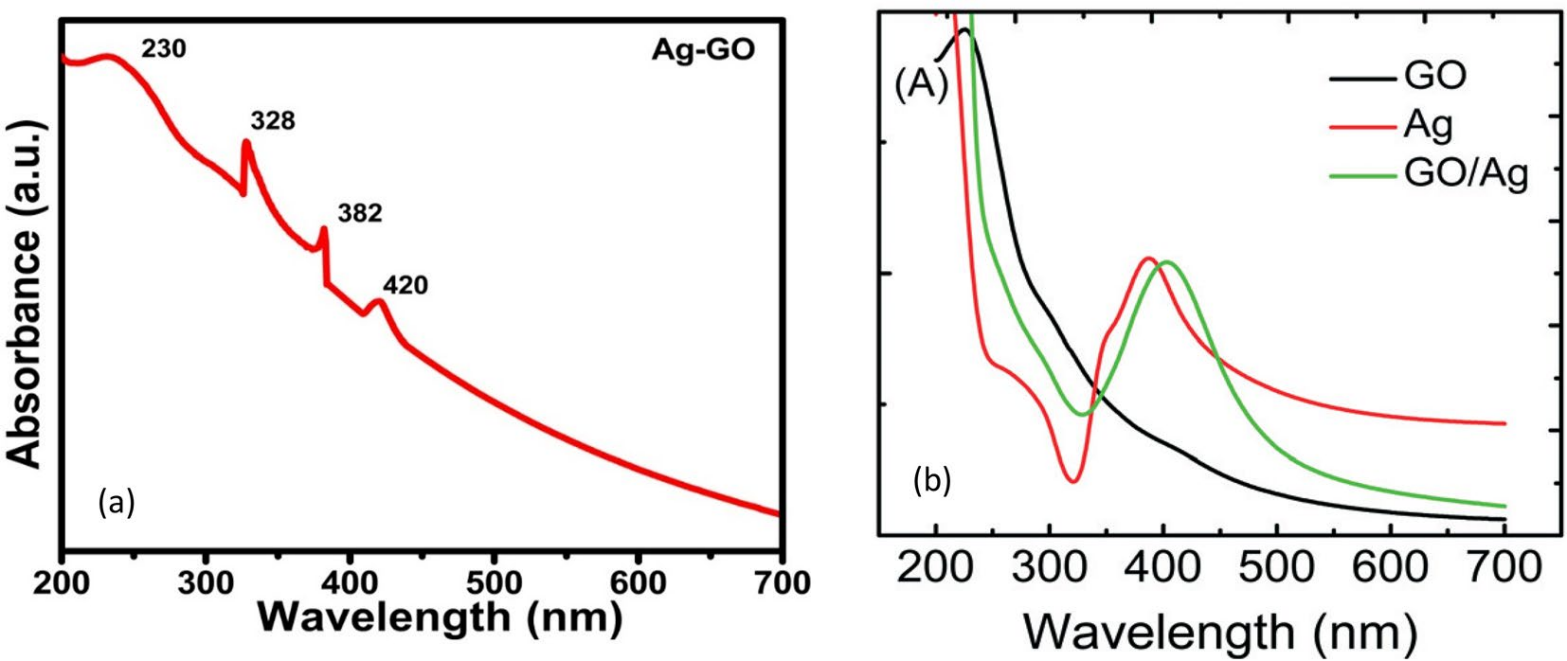
(**a**) UV–Vis spectra of Ag–GO sample (**b**) UV–Vis spectra of GO and Ag–GO reported in Ref.^17^.

Two different characteristic peaks were observed in the absorbance spectra of graphene oxide assigned to different transitions (see Fig. 1), which is in agreement with previous reports on graphene oxide^16^. First prominent absorption peak at 227 nm representing the conjugated π-π^*^ transitions of C=C bond of aromatic sp^2^ clusters, C–O that remain from the original graphite structure and the second one around 300 nm which claims to carbonyl (C=O) n-π^*^ transitions or due to the presence of epoxide (C–O–C) and peroxide functional groups (R–O–O–R) with α, β-unsaturations^17,18^. The absorption peak at 227 nm is compatible with the anticipated structure of the density function computation and reasonable compared to the UV–vis spectra of graphene. The blue shift in the graphene oxide UV–vis spectra is due to the constraint electronic conjugation increasing the LUMO and HUMO disturbance^19,20^. The π-π^*^ transitions can be due to two types of conjugative effect, first the nanometer-level sp^2^ cluster and second form due to the linking chromophore units as C=C, C=O, C–O bonds. Absorbance at the wavelength of 328 nm, a characteristic band of GO, was assigned to n → π* transitions of the carbonyl groups. The higher the intensity at ~ 300 nm, the larger the degree of oxidation of GO. Therefore, the absorption band at ~ 300 nm could be used as an indicator to estimate the degree of oxidation of GO. A peak at 420 nm can be referred to the silver nanoparticles on the GO sheets which is in agreement with the results as reported by Soroush et al. and is shown in Fig. 1b^17^. The other peaks which are appearing at 328 nm and 382 nm can be attributed to the graphene nano sheets. By keeping in mind all the above facts the change in UV–Vis absorption spectra of graphene oxide can be referred to the conjugative effect of the chromophore aggregation, that govern the *π*-π* transition^20^.

### FTIR spectroscopy

**Figure 2:**
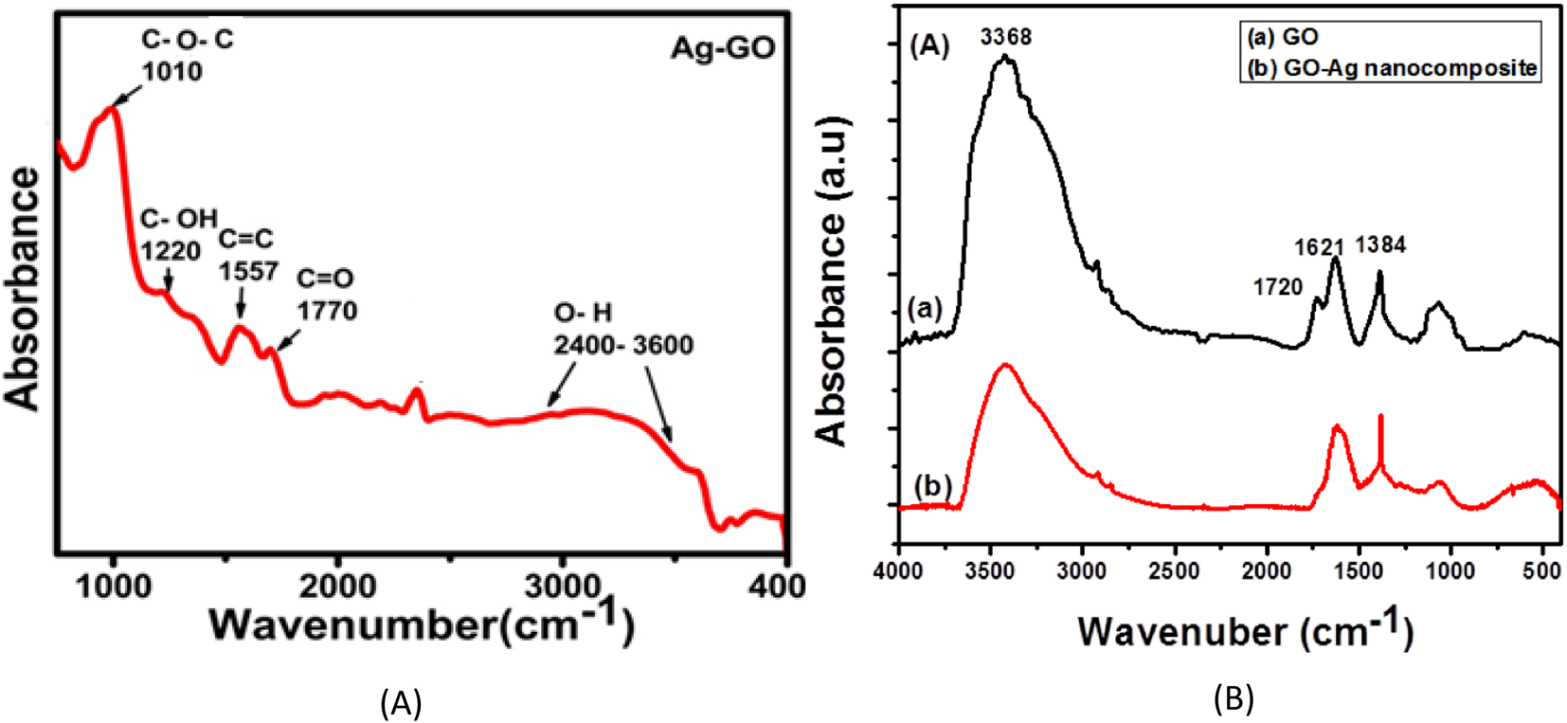
(**A**) FTIR spectrum of Ag–GO sample (**B**) FTIR spectra of GO and Ag–GO reported in Ref.^22^.

Figure 2a shows the FTIR spectra of Ag–GO that confirm the oxidation of graphite to graphene oxide (GO) having different oxygen containing functional groups. First peak in between 2500 cm^−1^ to 3650 cm^−1^ corresponds the O–H (Hydroxyl) stretching vibrations^21^. The more general attenuation of the hydrogen bonded O–H bands, within the region above 3300 cm^−1^, may be related to reduced water content in the Ag–GO and/or interaction of the silver with the hydrogen bonded O–H groups. Appearance of peaks in between 1707 cm^−1^ to 1742 cm^−1^ shows the stretching vibration of the carbonyl or carboxyl groups and peak 1588 cm^−1^ to 1650 cm^−1^ refers to the C=C (skeletal vibrations of the oxidized graphitic domains)^22,23^. Khalil et al. reported FTIR spectra of GO and Ag–GO nanocomposites which also indicated that there is no significant change in GO and Ag–GO FTIR spectra (Fig. 2b). However it can be inferred that the more general attenuation of the hydrogen bonded O–H bands, within the region above 3300 cm^−1^ in our results may be related to reduced water content in the Ag–GO and/or interaction of the silver with the hydrogen bonded O–H groups. Moreover, the peaks are observed to be slightly shifted in our results. A comparison of FTIR peaks in this work and reference^22^ is given in Table 1.

**Table 1:** FTIR peaks of Ag–GO sample.

Stretching vibration	FTIR peaks (cm^−1^)	
	This work	Ref.^22^
O–H	2500–3650	3368
C–O–C	1010	1067
C–OH	1220	1387
C=C	1557	1621
C=O	1770	1720

XRD data discussed ahead, supports these observations which indicated that the usual relatively ordered structure of the GO had been disturbed by its interaction with the silver. It may be that the silver particles had intercalated the layers resulting in non-uniformity of stacking. This would lead to the significantly attenuated and broadened GO related reflections in the XRD data for the Ag–GO. A significant intensity decrease is observed in the peaks correspond to oxygenated functional groups of as prepared Ag–GO sample which is attributed to the presence of silver atoms on the GO sheets.

### Structural analysis

**Figure 3:**
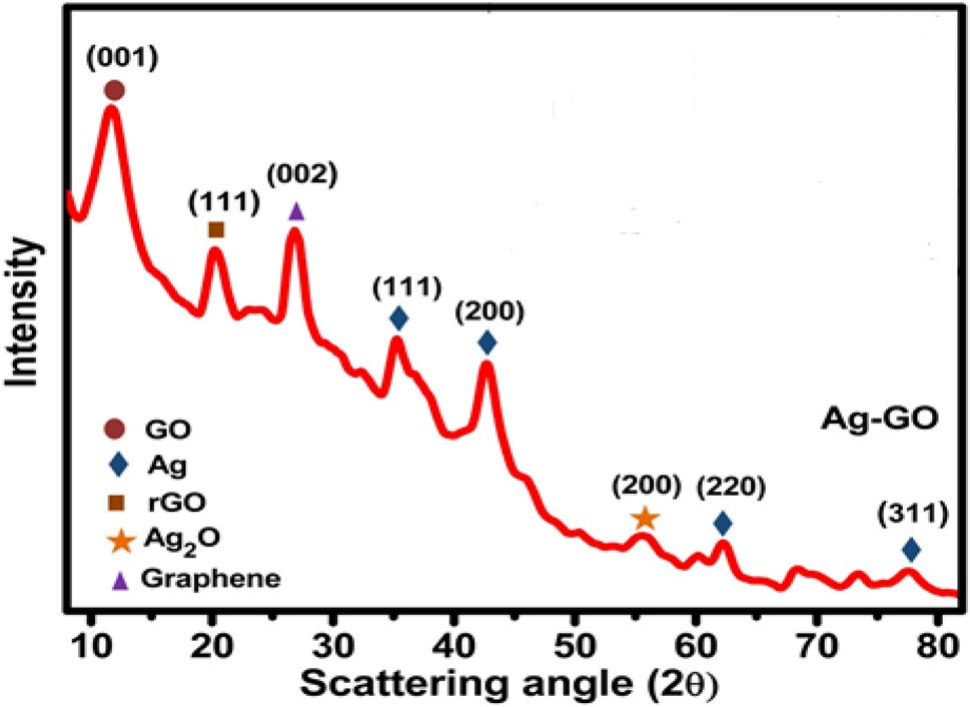
XRD pattern of Ag–GO sample.

X-ray diffraction is a technique which is used to characterize the crystallinity and the average grain size and the d-spacing between the planes. Figure 3 shows the XRD pattern of silver-graphene oxide. XRD spectra show a strong and prominent peak appear at 2-theta 11.5° possessing (001) plane having d-spacing 0.384 nm assure the formation of GO and also peak at about 21° represents the graphitic planes with minimal oxygen functional groups and in the XRD diffractogram labelled as reduced graphene oxide (rGO). As typical GO exhibits interlayer spacing ~ 0.83 nm and that the addition of the oxygen functional groups will result in increase in d-spacing^23–25^. A peak appears at the 2-theta of 26.7° having the plane (002) and d-spacing is 0.34 nm might be attributed to the short range order restacked graphene sheets^26^. This is consistent with FTIR spectra analysis. The diffraction peaks appear at 38°, 44°, 64°, and 77° represent the crystallographic planes of (111), (200), (220), and (311) for the face-centered cubic of the silver crystal and well matched with the standard X-ray diffraction (XRD) pattern (JCPDS no. 04-0783).

### Raman spectroscopy

**Figure 4:**
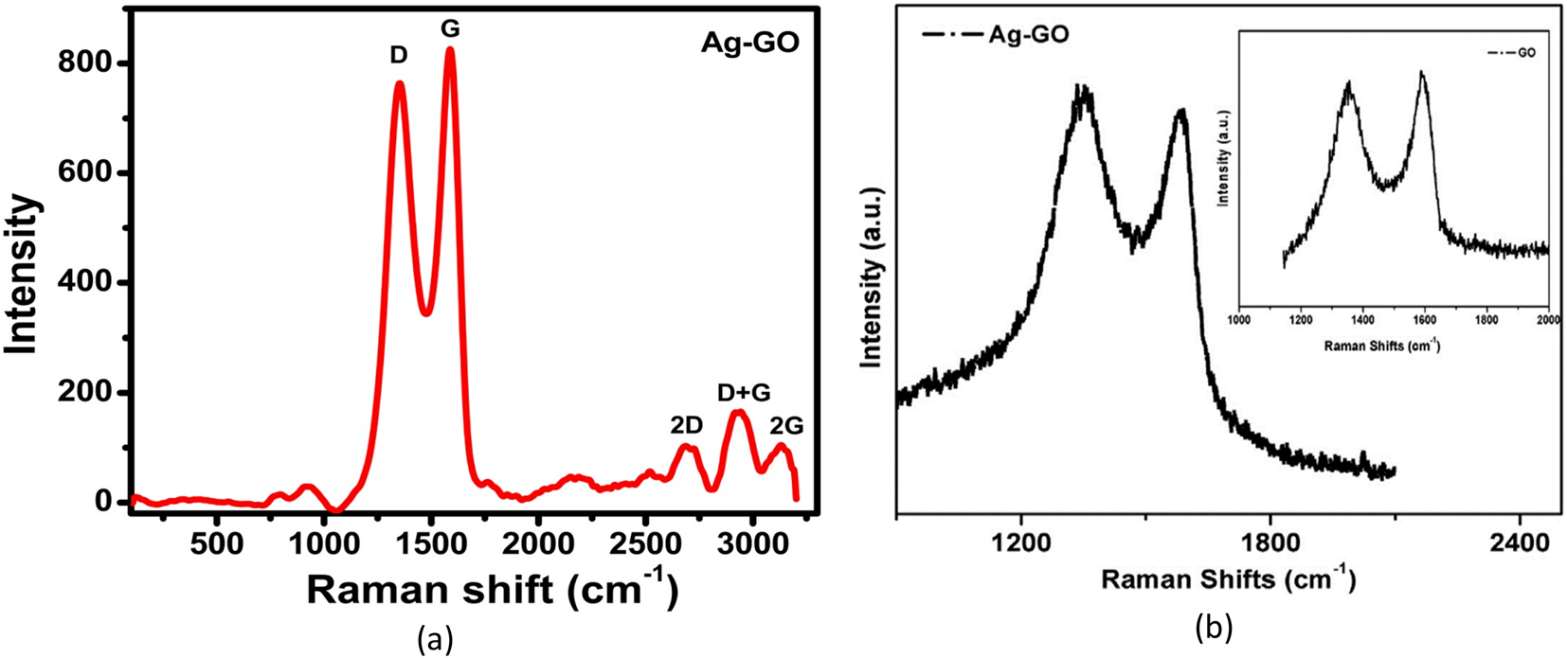
(**a**) Raman spectrum of Ag–GO sample (**b**) Raman spectra of GO and Ag–GO nanocomposite reported in reference^35^.

Raman spectroscopy is widely used technique to characterize the carbon-based products, because the conjugated or double bonded carbon atoms corresponds to great Raman intensities. In the case of graphene oxide (GO) this technique is useful to determine quality, number of layers and their arrangements, crystallite size and defects as well^27,28^. Raman spectrum of Ag–GO is depicted in Fig. 4. The first peak known as D-band which is at 1352 cm^−1^, originate from the TO phonons nearby the Dirac points (K) of the Brillouin zone and mainly associated to the structural defects or distortions in the graphitic planes/domains like grain boundaries, bond length disorder, vacancies and alien atoms in the graphene planes^29^. Since D-band depends on the oxygen content in GO, decrease in D-band intensity could be the possible reason of minimal oxygen functional groups due to the presence of Ag nanoparticles. The peak corresponds to G-band is observed at 1595 cm^−1^ due to the in phase first order Raman scattering of the E_2g_ phonons by their sp^2^-hybridized carbon at the Brillouin zone center and related to stretching of graphite lattice^30^. The intensities of the D and G bands are 820 and 969 cm^−1^ and the defect ratio (I_D_/I_G_) of is 0.846 confirms that the oxygenated functional groups (carboxylic, epoxide and hydroxyl) invade into the graphitic domain^31^.

In addition to this spectrum overtone bands 2D, D + G ~ G_0_ and 2G are also observed at 2688, 2952, 3140 cm^−1^ respectively which are highly effected by the oxidation of the graphitic structure^32,33^. As the ratio of the two bands I_2D_/I_G_ is 0.11 and it can be referred to multilayer graphene oxide. If we talk about the single layer graphene, then there is no significant D peak in the Raman spectrum and the width of this peak is related with the number of layers. The ratio of the intensity of the G-band to the D-band is related to the in-plane crystallite size according to Tuinstra-Koenig relation^34^ and calculated to be 5.13 nm. Moharana et al. studied Ag–GO nanocomposites and reported the Raman spectra of GO and Ag–GO Fig. 4b^35^. They reported D and G band at 1356 cm^−1^ and 1600 cm^−1^ for GO with a slight shift in Ag–GO nanocomposite and they attributed this shift to the interaction between Ag nanoparticles and GO. The Raman spectrum observed in this work is in agreement with^35^ and confirms the formation of Ag–GO nanocomposite.

$${L}_{a} (nm)= 4.4\left(\frac{{I}_{G}}{{I}_{D}}\right)$$

### AFM analysis

**Figure 5:**
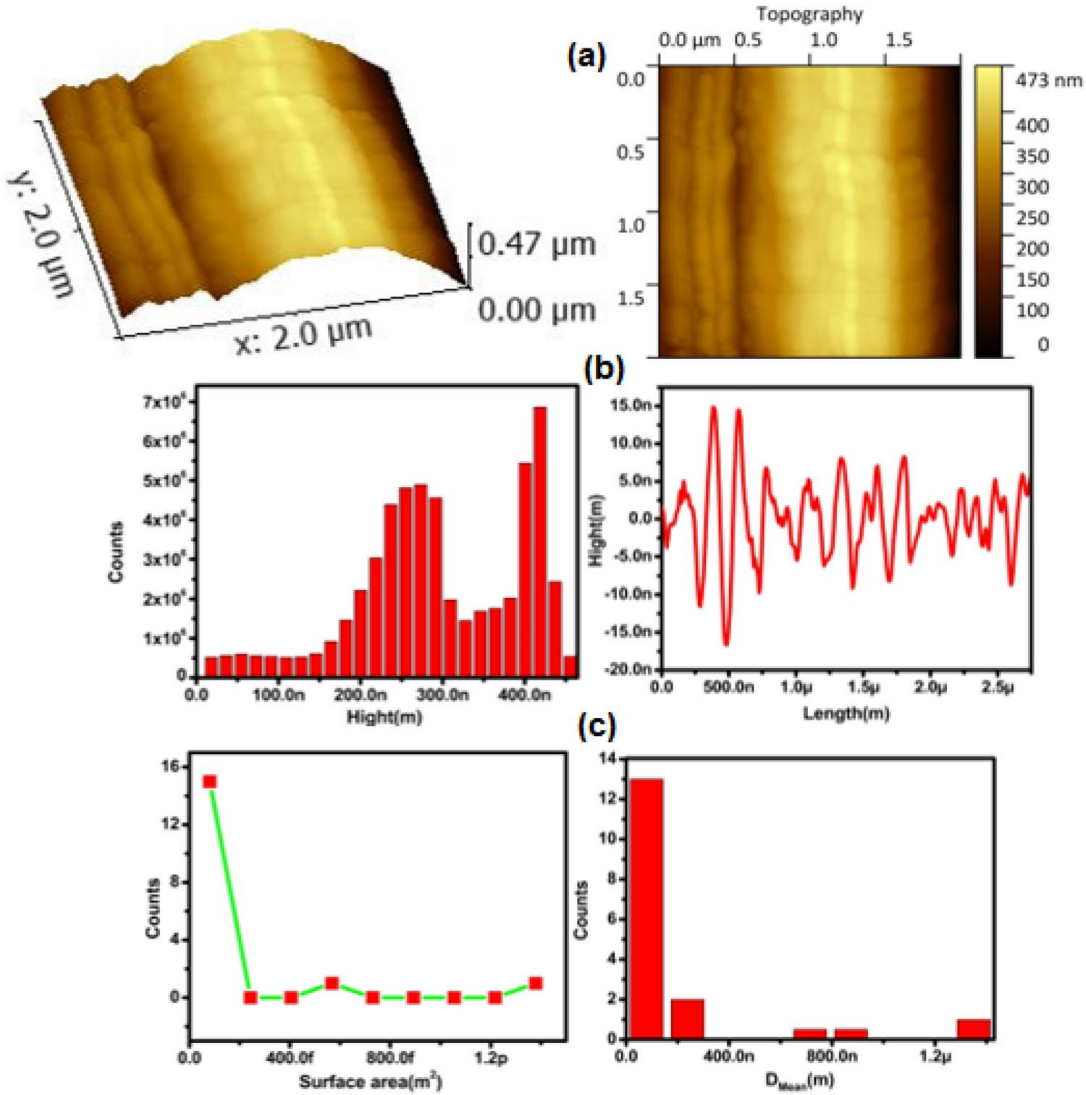
AFM topography images (**a**), Height distribution and height profile (**b**), surface area and particle size distribution (**c**) of Ag–GO sample.

AFM analysis of the Ag–GO sample was done using Atomic force microscopy (XE7-Park Systems) and shown in Fig. 5.The AFM image indicates a wavy structure formed due to wrinkles which can be clearly seen in the 3D topography image of the sample. In addition to the wrinkles, grain boundaries are also observed in the structure. The mean particle size, roughness, surface area, grain density and height profile of the sample were calculated using AFM software and listed in Table 2 while the graphs are shown in Fig. 5. All the graphs indicate an inhomogeneous distribution of grains, surface area and height responsible for the roughness of the Ag–GO sample. Mean grain diameter was found to be 151 nm while mean square roughness of the sample is 5.3 nm and the mean square waviness was 104 nm that is due to wrinkled graphene oxide.

**Table 2:** Grain size, roughness and grain density.

Sample	Mean grain size, D_mean_ (nm)	Root mean square roughness, R_q_ (nm)	Maximum height, nm	Root Mean square waviness, W_q_ (nm)	Grain density No. of grains/µm^2^
Ag–GO	151	5.3	22.2	104	9.18

### SEM analysis

**Figure 6:**
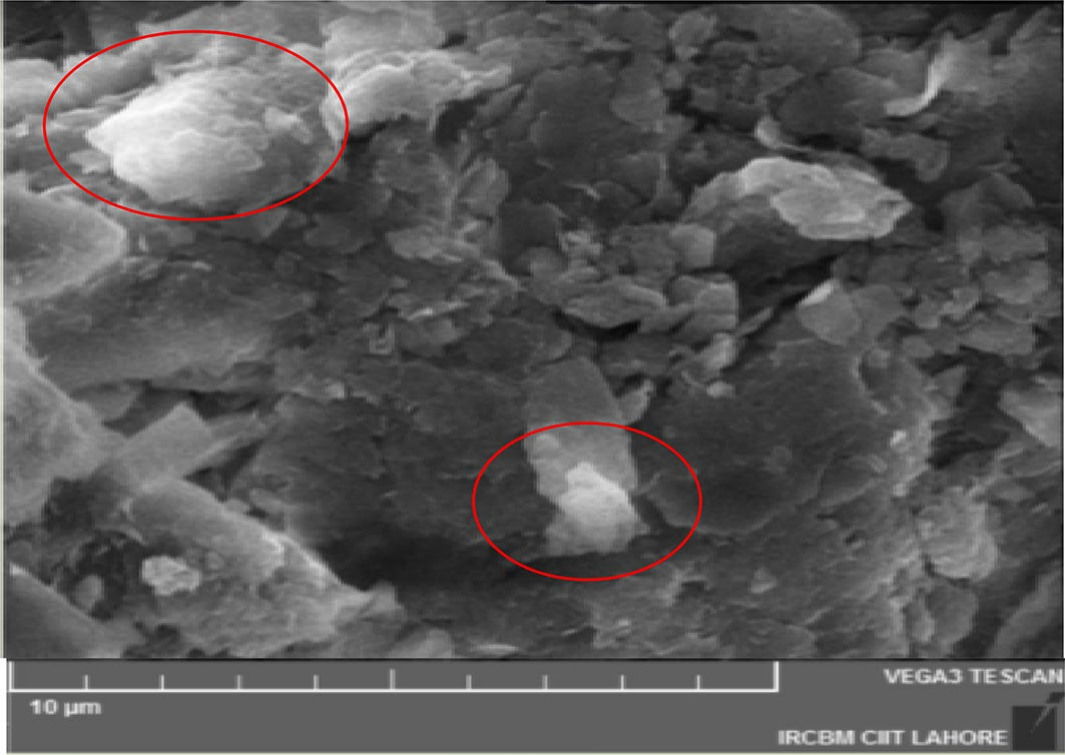
SEM micrograph of Ag–GO nanocomposite.

The SEM images of the Ag–GO sample recorded using SEM Tescan VEGA-3LMU. SEM image exhibits the crumbled sheets of GO decorated with light colored Ag Nano particles (see Fig. 6). The Ag nano particles are observed to be distributed throughout the GO sheet. The result showed that the GO sheet acted as a substrate to stabilize the aggregation of AgNPs.

### EDX analysis

**Figure 7:**
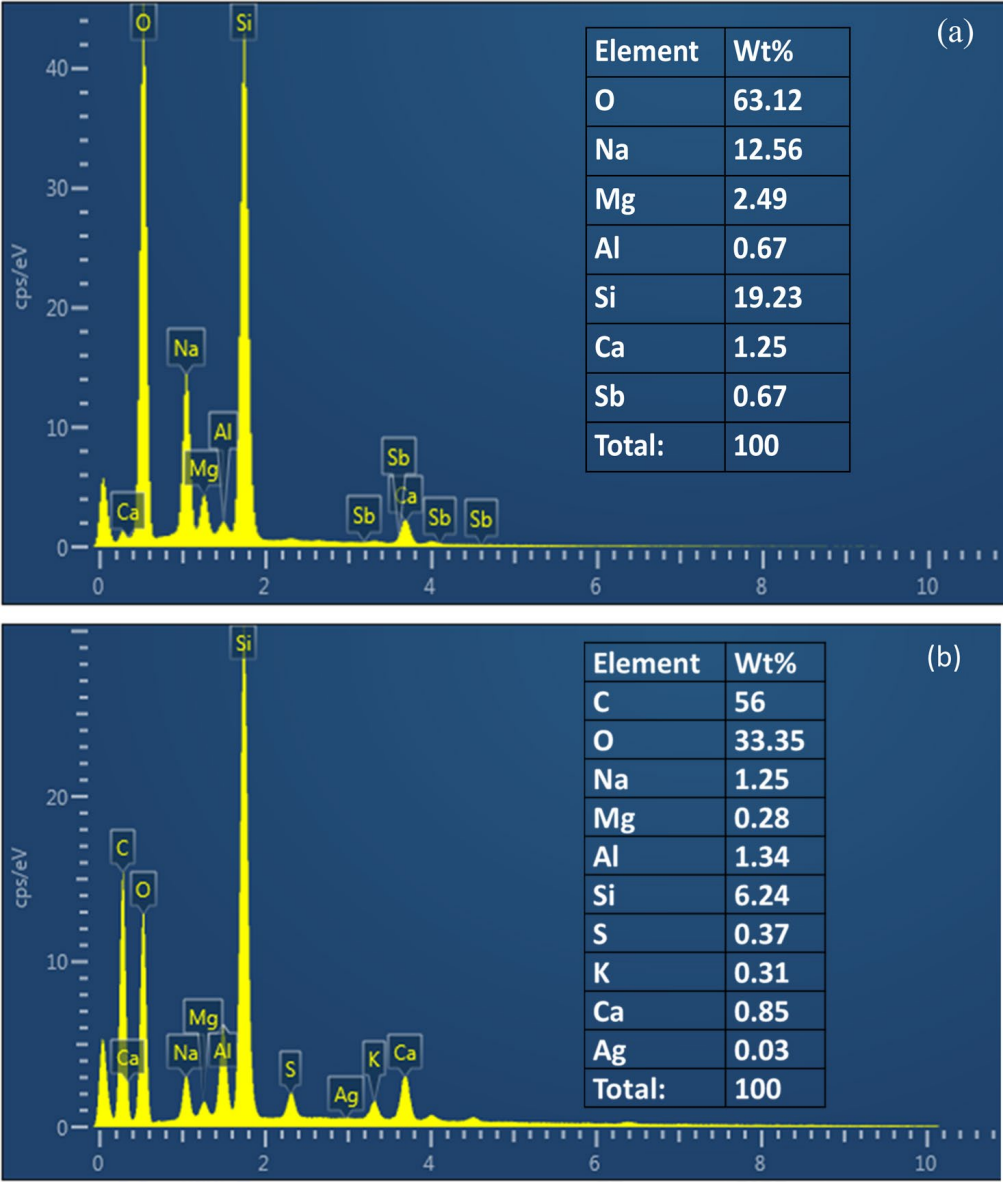
SEM micrograph of Ag–GO nanocomposite.

The EDX spectra (Fig. 7) of the bare glass (Fig. 7a) and that of Ag–GO sample (Fig. 7b) (as the sample was deposited on glass substrate using drop casting for characterization purpose). The EDX spectra also corroborated the presence of different spectral signals or carbon (C) with weight percentage 56% and oxygen (O) with weight percentage 33.35%^36^. The Ag with very low weight percentage 0.03% was detected which appears to be masked by Si peak which can be attributed to the glass substrate.

### Antibacterial test

The antibacterial activity of the Ag–GO sample was evaluated by using commonly found in hospital environment, gram-negative bacteria (*E. coli*) in LB-Broth via turbidity method and depicted in Fig. 7. From the figure it can be seen that Ag–GO exhibited strong antibacterial activity towards *E coli*. Percentage growth of *E. coli* was calculated by measuring optical density (O.D) values of the media and *E. coli* against low (100 μl) and high (300 μl, 500 μl) concentration after 24 h and 48 h time. The maximum percentage growth of *E. coli* in LB-Broth medium in an incubator (shaken at 150 rpm at 37 °C) is recorded as 95% after 24 h and 94.98% after 48 h in the absence of Ag–GO as shown in Table 3.

**Table 3:** Antibacterial activity effects of Ag–GO on *E. coli.*

Pathogenic bacterium	After 24 h incubation			After 48 h incubation		
	OD value for culture $\text{R}$	OD value for culture + media $\text{S}=\text{R}+0.086$	% Growth $\frac{R}{S}\times 100$	OD value for culture $\text{R}$	OD value for culture + media $\text{S}=\text{R}+0.088$	% Growth $\frac{R}{S}\times 100$
*E. coli*	1.650	1.736	95.04%	1.66	1.748	94.96%

Sample name/dose	OD Value for culture $\text{U}=\text{culture OD}-\text{sample OD}$	OD value for culture + media $\text{V}=\text{U}+0.086$	% Growth $\frac{U}{V}\times 100$	OD Value for culture $\text{U}=\text{culture OD}-\text{sample OD}$	OD value for culture + media $\text{V}=\text{U}+0.088$	% Growth $\frac{U}{V}\times 100$
Ag–GO (100 μl)	0.096–0.038 = 0.058	0.144	40.27%	0.051–0.038 = 0.013	0.101	12.87%
Ag–GO (300 μl)	0.120–0.038 = 0.082	0.168	48.80%	0.112–0.038 = 0.074	0.162	45.67%
Ag–GO (500 μl)	0.147–0.038 = 0.109	0.195	55.89%	0.131–0.038 = 0.093	0.181	51.38%

Compared to the control sample, Ag–GO shows high antibacterial activity as only few *E. coli* cells (~ 40%) were able to form colonies after 24 h and no more cells (~ 12%) viable after 48 h at low concentration. This indicated that the Ag–GO sample adsorbed on to the cell surface which prevented the bacteria nourishing. Because bacteria cells wrapped by Ag–GO were biologically disconnected from their cultural environment that blocks the cells’ access to nutrients and consequently they could not proliferate in culture medium. Besides, the destructive power of Ag–GO to cell membrane increases because Ag NPs could contact the cell membrane and eventually damage the cell. The Ag–GO is supposed to wrap the treated bacteria cell that facilitates Ag NPs to develop a direct contact with the cell membrane^36,37^.

**Figure 8:**
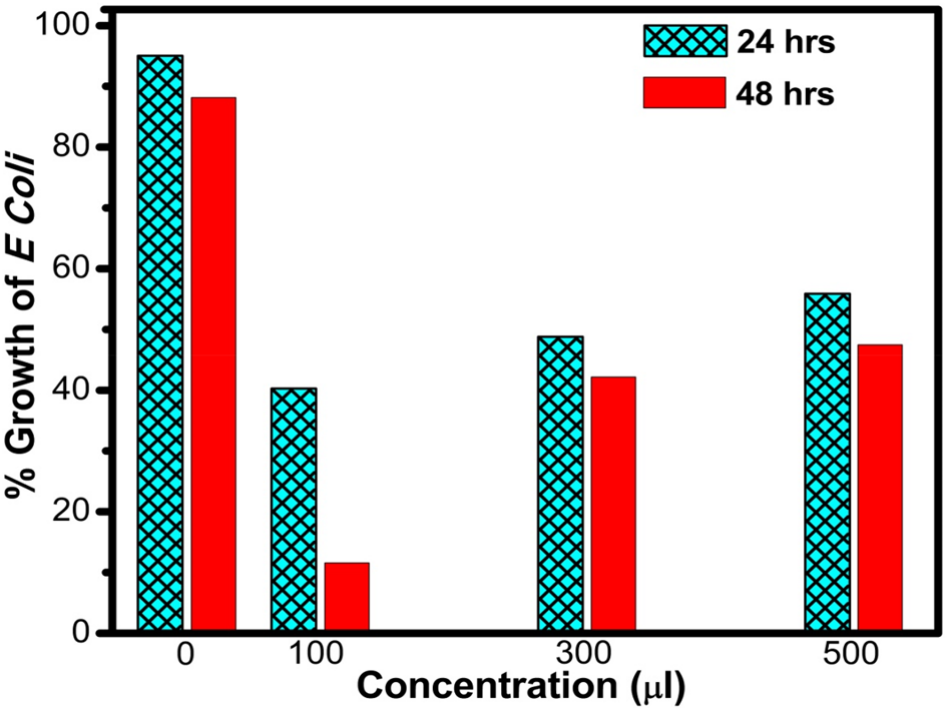
Percentage growth of *E. coli* treated with different concentrations of Ag–GO nanocomposite.

This attachment is attributed to the strong electrostatic interaction between gram negative bacterium (*E. coli*) and Ag–GO because both GO and *E. coli* are negatively charged which significantly decreases in the presence of Ag NPs. This screening of the surface charge increases the probability of bacteria cells being contacted with Ag–GO thus improving the antimicrobial properties of the Ag–GO. While for high concentration it was observed that there were still plenty of bacteria cells (~ 55%) survived after being treated with 24 h and (~ 51%) survived after 48 h^38^ as shown in Fig. 8. However, generally it is expected that Ag^2+^ exhibits stronger inhibition at higher concentrations which is not the case in our experiment. One possible factor could be the agglomeration and aggregates of the Ag^2+^ nanoparticles which resulted in an increase in particle size hence transforming the nanoparticles into different morphological structures such as, spheres, rods, discs etc. eventually it decreased the surface area and hence the reactivity.

The agglomeration of NPs in cell culture media may alter their physicochemical properties and colloidal behavior that effects their cellular association and cytotoxicity. Moreover, the reduced surface area minimized the contact between the bacteria cells and the agglomerated nanoparticles thereby decreasing their antibacterial activity at higher concentrations of the dose. Since, it is well known that decreasing the size of Ag NPs increases the antimicrobial activity due to the increased surface area, which promotes higher interaction between NPs and cell membrane. It is therefore confirmed that the as synthesized surfactant free Ag–GO exhibited antibacterial properties that inhibited the growth of gram-negative *E. coli*. The pivotal role was played by the oxidation of the graphitic planes and Ag^2+^ nanoparticles, which were responsible for the oxidation of the outer and inner membranes of the bacterial cell which were eventually destructed and released their vital material like adenine and protein, which ultimately caused cell’s death^39^.

## Conclusion

In summary, we report a facile one step route of Ag–GO nanocomposite formation excluding the aid of surfactants and reductants and observed synergistic antibacterial activity against negative *Escherichia coli* (*E. coli*) as a model. The bacteria growth rate was inhibited up to 40.27% with the lowest doze concentration of 100 μl in first 24 h of the antibacterial test indicating its efficient antibacterial activity which decreased with increasing doze concentration. The formation of Ag–GO nanocomposite was confirmed using Fourier transform infrared spectroscopy (FTIR) and Raman Spectroscopy. FTIR results confirmed the existence of oxygen-containing functional groups and found to be in consistence with Raman spectra. The detailed structural properties were investigated by X-ray diffraction (XRD) and observed the successful formation of GO (peak @$2\theta \sim 11.5$) mediated with silver nanoparticles. The absorption spectra (peak ~ 300 nm) showed clear evidence of the degree of oxidation the presence of silver nanoparticles (peak ~ 420 nm) over GO nanosheets due to surface plasmon resonance. Atomic Force Microscopy (AFM) and SEM images showed Ag nanoparticles decorated crumbled GO sheets with wrinkles. In conclusion, the successful formation of polymer or surfactant mediator free Ag–GO nanocomposite was achieved with a one step and facile synthetic route and it can be suggested from that surfactant free Ag–GO can be efficiently applied for antibacterial applications while reducing chemical reagents biological and environmental implications involved otherwise.

## References

[1] Oscar N, Fernando R, Baojiang KAS, Nicholas W, Pengju AB, Nicholas GL, Marlin DM, Ya-Ping V, Christopher EB (2011). Graphene oxide: A nonspecific enhancer of cellular growth. ACS Nano.

[2] Pasricha R, Gupta S, Srivastava AK (2009). A facile and novel synthesis of Ag–graphene-based nanocomposite. Small..

[3] Chung C, Kim YK, Shin D, Ryoo SR, Hong BH, Min DH (2013). Biomedical applications of graphene and graphene oxide. Acc. Chem. Res..

[4] Sims CM, Hanna SK, Heller DA, Horoszko CP, Johnson ME, Bustos ARM, Reipa V, Riley KR, Nelson BC (2017). Redox-active nanomaterials for nanomedicine applications. Nanoscale.

[5] Szunerits S, Boukherroub R (2016). Antibacterial activity of graphene based materials. J. Mater. Chem. B.

[6] Zhao R, Lv M, Li Y, Sun M, Kong W, Wang L, Song S, Fan C, Jia L, Qiu S, Sun Y, Song H, Hao R (2017). Stable nanocomposite based on PEGylated and silver nanoparticles loaded graphene oxide for long-term antibacterial activity. ACS Appl. Mater. Interface.

[7] Neppolian B, Wang C, Ashokkumar M (2014). Sonochemically synthesized mono and bimetallic Au–Ag reduced graphene oxide based nanocomposites with enhanced catalytic activity. Ultrason. Sonochem..

[8] Cheng CE, Tsai CW, Pei Z, Lin TW, Chang CS, Chien FSS (2015). UV-treated graphene oxide as anode interfacial layers for P3HT: PCBM solar cells. J. Phys. D Appl. Phys..

[9] Aslam S, Mustafa F, Ahmad MA, Idrees M, Saleem M, Bhatti AS (2014). Photovoltaic performance and impedance spectroscopy of ZnS-Cu–Go nanocomposites. Ceram. Int..

[10] Wan Y, Wang Y, Wu J, Zhang D (2011). Graphene oxide sheet-mediated silver enhancement for application to electrochemical biosensors. Anal. Chem..

[11] Rasoulzadehzali M, Namazi H (2018). Facile preparation of antibacterial chitosan/graphene oxide–Ag bio-nanocomposite hydrogel beads for controlled release of doxorubicin. Int. J. Biol. Macromol..

[12] Zhao R, Kong W, Sun M, Yang Y, Liu W, Lv M, Song S, Wang L, Song H, Hao R (2018). Highly stable graphene-based nanocomposite (GO–PEI–Ag) with broad-spectrum, long-term antimicrobial activity and antibiofilm effects. ACS Appl. Mater. Interfaces.

[13] Naeem H, Ajmal M, Qureshi RB, Muntha ST, Farooq M, Siddiq M (2019). Facile synthesis of graphene oxide–silver nanocomposite for decontamination of water from multiple pollutants by adsorption, catalysis and antibacterial activity. J. Environ. Manag..

[14] Mao A, Zhang D, Jin X, Gu X, Wei X, Yang G, Liu X (2018). Synthesis of graphene oxide sheets decorated by silver nanoparticles in organic phase and their catalytic activity. J. Phys. Chem. Solids.

[15] Fathalipour S, Mardi M (2017). Synthesis of silane ligand-modified graphene oxide and antibacterial activity of modified graphene–silver nanocomposite. Mater. Sci. Eng. C.

[16] Marcano DC, Kosynkin DV, Berlin JM, Sinitskii A, Sun Z, Slesarev A, Alemany LB, Wei L, James MT (2010). Improved synthesis of graphene oxide. ACS Nano.

[17] Soroush A, Ma W, Silvino Y, Rahaman MS (2015). Surface modification of thin film composite forward osmosis membrane by silver-decorated graphene-oxide nanosheets. Environ. Sci. Nano.

[18] Zhang Y, Ma HL, Zhang Q, Peng J, Li J, Zhai M, Yu JJ (2012). Facile synthesis of well-dispersed graphene by γ-ray induced reduction of graphene oxide. J. Mater. Chem..

[19] Saxena S, Tyson TA, Shukla S, Negusse E, Chen H, Bai J (2011). Investigation of structural and electronic properties of graphene oxide. Appl. Phys. Lett..

[20] Lai Q, Zhu S, Luo X, Zou M, Huang S (2012). Ultraviolet-visible spectroscopy of graphene oxides. AIP Adv..

[21] Paulchamy B, Arthi G, Lignesh BD (2015). A simple approach to stepwise synthesis of graphene oxide nanomaterial. J. Nanomed. Nanotechnol..

[22] Khalil WA, Sherif HH, Hemdan BA, Khalil SK, Hotaby WE (2019). Biocompatibility enhancement of graphene oxide–silver nanocomposite by functionalisation with polyvinylpyrrolidone. IET Nanobiotechnol..

[23] Hasan V, Senger BJ, Mulford P, Ryan C, Doan H, Gryczynski Z, Naumov AV (2017). Modifying optical properties of reduced / graphene oxide with controlled ozone and thermal treatment in aqueous suspensions. Nanotechnology.

[24] Chook SW, Chia CH, Zakaria S, Ayob MK, Chee KL, Huang NM, Neoh HM, Lim HN, Jamal R, Rahman RA (2012). Antibacterial performance of Ag nanoparticles and AgGO nanocomposites prepared via rapid microwave-assisted synthesis method. Nanoscale Res. Lett..

[25] Rani JR, Lim J, Oh J, Kim D, Lee D, Kim JW, Shin HS, Kim JH, Jun SC (2013). Substrate and buffer layer effect on the structural and optical properties of graphene oxide thin films. RSC Adv..

[26] Gurunathan S, Han JW, Kim JH (2013). Green chemistry approach for the synthesis of biocompatible graphene. Int. J. Nanomed..

[27] Contreras JG, Briones FC (2015). Graphene oxide powders with different oxidation degree, prepared by synthesis variations of the Hummers method. Mater. Chem. Phys..

[28] Low FW, Lai CW, Hamid SBA (2015). Easy preparation of ultrathin reduced graphene oxide sheets at a high stirring speed. Ceram. Int..

[29] Yu H, Zhang B, Bulin C, Li R, Xing R (2016). High-efficient synthesis of graphene oxide based on improved hummers method. Sci. Rep..

[30] El-Khodary SA, El-Enany GM, El-Okr M, Ibrahim M (2014). Preparation and characterization of microwave reduced graphite oxide for high-performance supercapacitors. Electrochem. Acta.

[31] Kaniyoor A, Ramaprabhu S (2014). A Raman spectroscopic investigation of graphite oxide derived graphene. AIP Adv..

[32] Krishnamoorthy K, Veerapandian M, Yun K, Kim SJ (2013). The chemical and structural analysis of graphene oxide with different degrees of oxidation. Carbon.

[33] Aslam S, Mustafa F, Ahmad MA (2018). Facile synthesis of graphene oxide with significant enhanced properties for optoelectronic and energy devices. Ceram. Int..

[34] Subrahmanyam KS, Vivekchand SRC, Govindaraj A, Rao CNR (2008). A study of graphenes prepared by different methods: Characterization, properties and solubilization. J. Mater. Chem..

[35] Moharana S, Mahaling RN (2017). Silver (ag)-graphene oxide (GO)—poly (vinylidene fluoride-co-hexafluoropropylene) (PVDF-HFP) nanostructured composites with high dielectric constant and low dielectric loss. Chem. Phys. Lett..

[36] Gao Y, Wu J, Ren X, Tan X, Hayat T, Alsaedi A, Cheng C, Chen C (2017). Impact of graphene oxide on antibacterial activity of antibiotics against bacteria. Environ. Sci. Nano.

[37] Ma J, Zhang J, Xiong Z, Yong Y, Zhao XS (2011). Preparation, characterization, and antibacterial properties of silver-modified graphene oxide. J. Mater. Chem..

[38] Jia T, Guo H, Zhang Q, Peng Q, Tang Y, Yan X, Li B (2015). Reduced graphene oxide-based silver nanoparticle-containing composite hydrogel as highly efficient dye catalysts for wastewater treatment. Sci. Rep..

[39] Nanda SS, Yi DK, Kim K (2016). Study of antibacterial mechanism of graphene oxide using Raman spectroscopy. Sci. Rep..

